# Supplementary material for: Luteolin and Vernodalol as Bioactive Compounds of Leaf and Root Vernonia amygdalina Extracts: Effects on α-Glucosidase, Glycation, ROS, Cell Viability, and In Silico ADMET Parameters
Source: Pharmaceutics. 2023 May 19;15(5):1541. doi: 10.3390/pharmaceutics15051541 (PMC10224510; doi:10.3390/pharmaceutics15051541)
Supplement: Supplementary file 1 [file pharmaceutics-15-01541-s001.zip › pharmaceutics-2338614-supplementary.pdf]

pharmaceutics

Article

Luteolin and Vernodalol as Bioactive Compounds of Leaf and Root *Vernonia amygdalina* Extracts: Effects on α-Glucosidase, Glycation, ROS, Cell Viability, and In Silico ADMET Parameters

Francine Medjiofack Djeujo, Valentina Stablum, Elisa Pangrazzi, Eugenio Ragazzi and Guglielmina Froldi *

Department of Pharmaceutical and Pharmacological Sciences, University of Padova, 35131 Padova, Italy; francine.medjiofackdjeujo@phd.unipd.it (F.M.D.); etwvalentina@gmail.com (V.S.); elisapangrazzi99@gmail.com (E.P.); eugenio.ragazzi@unipd.it (E.R.)

* Correspondence: g.froldi@unipd.it; Tel.: +39-049-827-5092; Fax: +39-049-827-5093

Citation: Djeujo, F.M.; Stablum, V.; Pangrazzi, E.; Ragazzi, E.; Froldi, G. Luteolin and Vernodalol as Bioactive Compounds of Leaf and Root *Vernonia amygdalina* Extracts: Effects on α-Glucosidase, Glycation, ROS, Cell Viability, and In Silico ADMET Parameters. *Pharmaceutics* **2023**, *15*, 1541. https://doi.org/10.3390/pharmaceutics15051541

Academic Editors: Jadwiga Renata Ochocka and Justyna Stefanowicz-Hajduk

Received: 27 March 2023
Revised: 15 May 2023
Accepted: 18 May 2023
Published: 19 May 2023

Copyright: © 2023 by the authors. Licensee MDPI, Basel, Switzerland. This article is an open access article distributed under the terms and conditions of the Creative Commons Attribution (CC BY) license (https://creativecommons.org/licenses/by/4.0/).

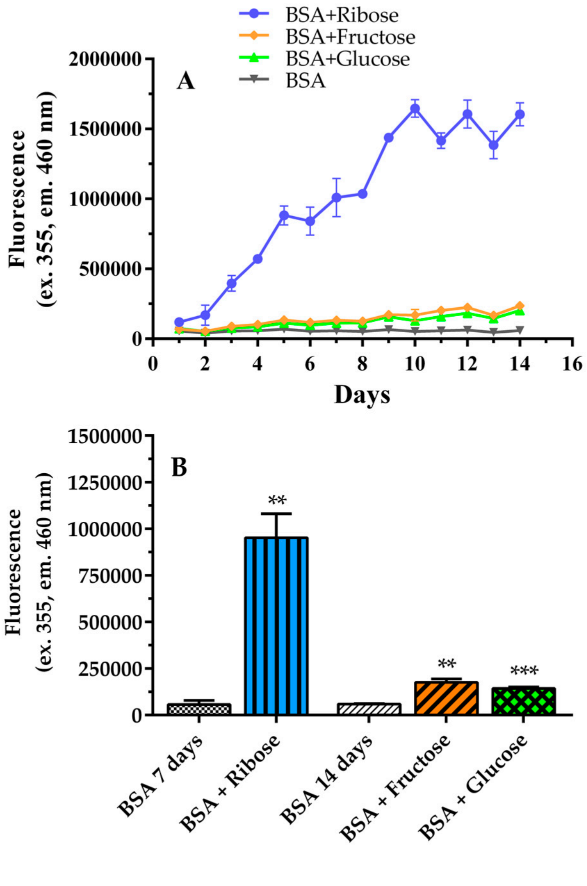

Figure S1. Kinetics of BSA glycation obtained with three different glycating agents, such as ribose, fructose and glucose. **: $p < 0.01$; *** $p < 0.005$ versus α-glucosidase activity.
